# Use of Flow Cytometry to Evaluate Phagocytosis of *Staphylococcus aureus* by Human Neutrophils

**DOI:** 10.3389/fimmu.2021.635825

**Published:** 2021-02-19

**Authors:** Elena Boero, Iris Brinkman, Thessely Juliet, Eline van Yperen, Jos A. G. van Strijp, Suzan H. M. Rooijakkers, Kok P. M. van Kessel

**Affiliations:** ^1^ Medical Microbiology, University Medical Center Utrecht, Utrecht University, Utrecht, Netherlands; ^2^ GlaxoSmithKline Vaccines S.r.l., Siena, Italy

**Keywords:** *Staphylococcus aureus*, human, neutrophils, phagocytosis, flow cytometry

## Abstract

Neutrophils play a key role in the human immune response to *Staphylococcus aureus* infections. These professional phagocytes rapidly migrate to the site of infection to engulf bacteria and destroy them *via* specialized intracellular killing mechanisms. Here we describe a robust and relatively high-throughput flow cytometry assay to quantify phagocytosis of *S. aureus* by human neutrophils. We show that effective phagocytic uptake of *S. aureus* is greatly enhanced by opsonization, i.e. the tagging of microbial surfaces with plasma-derived host proteins like antibodies and complement. Our rapid assay to monitor phagocytosis can be used to study neutrophil deficiencies and bacterial evasion, but also provides a powerful tool to assess the opsonic capacity of antibodies, either in the context of natural immune responses or immune therapies.

## Introduction

*Staphylococcus aureus* is a leading pathogen causing an array of serious and possibly fatal diseases in humans (1). Due to its fast acquisition of antibiotic resistance, treatment of *S. aureus* infections is becoming increasingly difficult (2). Alternative measures such as vaccine candidates tested so far were unsuccessful (3, 4), thus new research efforts are urgently required. Patients with compromised neutrophil function are more susceptible to *S. aureus* infections (5). This suggests that studying the role of neutrophils in the clearance of different clinical strains of this bacterium could provide us with clues to develop therapies.

Neutrophils are the most abundant professional phagocytes of the innate immune system. These terminally differentiated cells are regularly released in the bloodstream to patrol the body. When *S. aureus* invades tissues, neutrophils sense inflammatory signals released by local cells and extravasate, migrating towards the site of infection (5–8). The inflammatory milieu also primes neutrophils, boosting their capacity to recognize and clear bacteria (9). Neutrophils mainly kill pathogens through phagocytosis, a process in which bacteria are engulfed into intracellular vesicles called phagosomes. The phagosomes then mature into lytic vesicles: they become filled with antimicrobial substances stored into cytoplasmic granules and with toxic reactive oxygen species. These events, combined with the tuning of the vacuolar pH, finely regulates the enzymatic activity, ultimately leading to killing and digestion of the bacteria (10, 11).

Phagocytosis is an active process that requires the direct contact between *S. aureus* and the neutrophil. This process is typically aided by opsonins, host plasma proteins that mark bacterial surfaces increasing their probability of interacting with neutrophils phagocytic receptors. The main opsonins are immunoglobulins and complement proteins (12).

Immunoglobulins are adaptive immune molecules designed to specifically recognize an antigen, such as Staphylococcal surface proteins. Once bound to the bacteria, immunoglobulins of class G (IgG) and A (IgA) induce phagocytosis by direct interaction of their Fc with their specific phagocytic receptors FcγRs and FcαRs. Furthermore, IgGs and IgMs induce phagocytosis indirectly, by activating the complement cascade *via* the classical pathway (13).

The complement system also comprises proteins that label bacterial surfaces that enhance phagocytosis. Since complement deposition is aspecific its activation is guided by three recognition pathways, including the aforementioned classical complement pathway in which complement activation is triggered by the recognition of *S. aureus* by antibodies (14, 15). The three recognition pathways all converge to the cleavage of C3 into C3b, which deposits on and tags bacterial surfaces. C3b is further inactivated into iC3b, and together they are ligands of neutrophil phagocytic receptors CR1 and CR3 (16).

In this methodological paper we describe an *in vitro* assay to measure phagocytosis. Traditionally, bacterial phagocytosis by human neutrophils is analyzed by enumeration of viable bacteria on growth media or by counting particles under the microscope. This method is very time-consuming since for each condition serial dilutions of lysed neutrophils are needed. Therefore, we and others have developed several approaches to determine phagocytosis of bacteria by neutrophils (17–30), many of them in multi-well plates. Here, we describe a powerful flow cytometry method to quantify phagocytosis of *S. aureus* by freshly isolated human neutrophils *in vitro*. Although our phagocytosis assay is not novel, we here intend to provide an extensive overview of the methodology, pinpoint basic parameters and considerations for the proper interpretation of results. In particular, we apply the method to unravel the role of different opsonins in the phagocytosis process using primary human neutrophils. By taking these parameters into account, a flow cytometric phagocytosis assay becomes a powerful method for studying neutrophil activity, as well as bacterial phenotypes and antibody responses.

## Materials and Methods

### Ethics Statement

Blood was obtained from healthy donors after informed consent was obtained from all subjects, in accordance with the Declaration of Helsinki. Approval from the Medical Ethics Committee of the University Medical Center Utrecht was obtained (METC protocol 07-125/C approved on March 1, 2010).

### *S. aureus* Strains Choice, Labeling and Culture

*S. aureus* strains used in phagocytosis assays were selected to have low or no expression of staphylococcal protein A (SpA) and second immunoglobulin-binding protein (Sbi), the main immunoglobulin binding proteins of *S. aureus*, that could otherwise interfere with opsonization and lower the resolution of the assay. In a separate experiment, several wild type *S. aureus* strains were included for comparison (see **Table 1**). All *S. aureus* strains were fluorescently labelled to allow detection by flow cytometry. We used both genetically labelled bacteria (GFP and mAm) and surface-labelled bacteria (FITC).

**Table 1:** *S. aureus* strains used.

*S. aureus* strains	Description	Reference or source	Notes
KV27	Clinical strain UMCU	Pleural effusion	Low SpA expression*
Newman *ΔSpA ΔSbi*	*spa sbi* knockout mutant	(31)	
Newman wt	Wild type strain	Tim Foster, Dublin	
MW2		Michael Otto, NIH	
8325-4		Tim Foster, Dublin	(32)
COL		Andreas Peschel, Germany	(32)
USA300		Frank Deleo, NIAID	(32)
Wood-46		ATCC10932	
SH1000		Tim Foster, Dublin	

*Determined by comparing isotype mouse IgG2a binding levels among several clinical S. aureus strains.

To construct GFP-labelled *S. aureus* strains, bacteria were transformed with a GFP-expressing plasmid pCM29 that constitutively and robustly expresses the superfolder green fluorescent protein (sGFP) from the sarAP1 promoter (33). A codon optimized gene for the fluorescent protein mAmetrine (mAm; GenBank: KX759016) was also cloned into the pCM29 plasmid (34). Competent bacteria (~5x10^9^) were electroporated with 10 µl plasmid with a Gene Pulser II (BioRad; 100 Ohm resistance, 25 µF capacitance at 2.5 kVolt) (35). After recovery, bacteria were plated on Todd Hewitt agar (THA) plates containing 10 µg/ml chloramphenicol. A colony was picked for propagation in liquid THB plus 10 µg/ml chloramphenicol, collected and stored in PBS + glycerol (final concentration 15%) at −80°C.

To prepare bacterial stocks for phagocytosis experiments, *S. aureus* was grown overnight in Todd Hewitt broth (THB) with the necessary antibiotics, diluted to an OD^660nm^ of 0.05 in fresh THB and cultured until an OD^660nm^ of ~ 0.50, corresponding to an early exponential phase. Bacteria were then collected by centrifuging at 2,400 x g at 4°C, for 15 min, washed twice, resuspended in RPMI + 0.05% HSA (RPMI-HSA), and stored aliquoted at −20°C.

For the chemical surface labeling with FITC, bacteria were grown as described above and after centrifugation labelled for 1 h at 4°C in 0.1 M carbonate buffer (pH 9.0) or PBS containing 250 µg/ml FITC. Finally, bacteria were washed thoroughly from excessive dye and stored at −20°C. Since the FITC isothiocyanate group reacts with amino terminal and primary amines available on the surface of the bacteria, we checked whether this procedure would affect the opsonization capacity of the serum. We therefore compared the phagocytosis of *S. aureus* expressing a mAmetrine fluorescent protein, unlabelled or externally labelled with FITC. The additional FITC labeling did not change the opsonization profile of the bacteria and did not interfere with phagocytosis (**Figure S1**).

### Isolation of Human Polymorphonuclear Leukocytes (PMNs)

PMNs were isolated from healthy donors’ blood. Whole blood was obtained by venepuncture and collected in sodium heparin 9 mL tubes (Greiner). Dual Ficoll/Histopaque density gradients were prepared by slowly layering 10 mL Ficoll (GE Healthcare) over 12 mL Histopaque (density 1.119; Sigma) in 50 mL tubes. Finally, 20–25 mL of blood diluted 1:1 (v/v) with PBS were carefully layered on top of the gradient, which was then centrifuged at 22°C for 20 min at 390 x g in a swinging bucket rotor without brake. After centrifugation the upper plasma, PBMC ring and the transparent layer of Histopaque were discarded. The pink Histopaque layer on top of the packed erythrocytes containing the PMNs was collected and washed with RPMI-HSA at 4°C for 10 min at 249 x g. Residual erythrocytes in the pellet were lysed by hypotonic shock with 9 mL of cold sterile deionized H_2_O. Osmolarity was restored with 1 mL of 10x PBS after exactly 30 s. Cells were washed again with RPMI-HSA at 4°C for 10 min at 249 x g and were suspended in RPMI-HSA. From 9 mL of blood the yield will range from 5 x 10^6^ to 3 x 10^7^, depending on the donor. For more details on the procedure see Surewaard et al. (30).

The isolated PMN fraction contains neutrophils, 1–5% non-phagocytosing eosinophils, other cell types and debris, with a viability of >97%.

### Preparation of Serum From Blood

Fresh blood was collected in tubes with no anticoagulant (Greiner) and allowed to clot undisturbed for 30 min at room temperature. The blood tubes were then centrifuged at 4°C for 10 min at 2,075 x g, and the supernatant (serum) was collected. Sera from 20 healthy individuals were stored individually at −80°C and pooled to be aliquoted as normal human serum (NHS), to avoid donor to donor variability in anti-staphylococcal antibody repertoire and complement activity. It is important to process the serum rapidly at low temperature and avoid freeze-thaw cycles to preserve the activity of complement. Heat-inactivated serum was prepared by incubating serum at 56°C for 30 min to deactivate thermosensitive complement proteins.

### Isolation of Antibodies From Serum and ΔIgGΔIgM NHS Production

The protocol is described in detail in by Zwarthoff (36). Briefly, part of the NHS was depleted from IgG and IgM (ΔIgGΔIgM NHS) by affinity chromatography, passing it through two affinity columns in tandem, a HiTrap Protein G High Performance column (GE Healthcare) and a Tricorn column filled with POROS CaptureSelect IgM Affinity Matrix (Thermo Scientific). The flow through fractions with serum-like appearance that also peaked in UV absorbance were pooled and stored aliquoted at −80°C. Roughly 70–80% of the original volume of serum was retrieved. Effectivity of the depletion of serum was determined by specific ELISAs leaving <1% of IgG and IgM. The residual complement activity of the ΔIgGΔIgM NHS was ~50%, evaluated by measuring the serum hemolytic activity of sheep erythrocytes (CH50).

IgG and IgM were separately recovered from their respective affinity columns using a 0.1 M glycine-HCl buffer at pH 2.7, runs pooled and dialyzed against PBS. Purified IgG and IgM were checked for purity on a size exclusion Superdex-200 column. NHS and ΔIgGΔIgM NHS were checked for the presence of IgG and IgM by ELISA using affinity purified Sheep-anti-Hu-IgG or anti-Hu-IgM respectively (ICN) as a capture and Peroxidase-labelled Goat-anti-Hu-IgG or anti-Hu-IgM (SouthernBiotech) as corresponding detection antibody. Purified IgG (Human Gamma Globulin; Jackson ImmunoResearch) or IgM (Sigma Aldrich) was used as reference.

### Classical Pathway Hemolytic Assay (CH50)

Sheep blood (bioTRADING Benelux B.V.) was washed twice with PBS to obtain pelleted red blood cells (ShRBC). ShRBC carry the Forssman glycolid structure and most humans (considered to be a Forssman antigen-negative species) have naturally occurring anti-Forssman antibodies (IgG and IgM) that would induce ShRBC lysis without additional opsonization. Therefore, human serum was pre-absorbed with pelleted ShRBC for 15 min on ice to eliminate these antibodies.

For the lysis experiment, pelleted ShRBC were resuspended in Veronal buffer (727 mM NaCl, 9.1 mM sodium barbital, 10.2 mM diethyl barbituric acid, pH 7.5) containing 0.5 mM CaCl_2_ and 0.25 mM MgCl_2_ and sensitized with 1:2,000 diluted Rabbit-anti-ShRBC serum for 10 min, washed and resuspended again in Veronal buffer. In a round-bottom 96-well plate, 25 µl of anti-Forssman antibodies-depleted human serum (starting at 10% with 2-fold dilutions) were mixed with 25 µl opsonized ShRBC and incubated for 60 min at 37°C on a shaking plateau. After centrifugation, release of hemoglobin was measured at 415 nm after transferring 25 µl supernatant to a half-area flat-bottom plate containing 25 µl MQ per well. Data were normalized for plateau 4% serum value (after subtraction of background spontaneous lysis) and used to calculate the EC50 value (GraphPad version 8; Sigmoidal dose–response with variable slope Least squares fit). EC50 is converted to titer using the formula 100/EC50.

### Affinity Purification of Anti-*S. aureus* IgGs

As a source of anti-*S. aureus* IgGs, we chose a commercial human normal immunoglobulin preparation for intravenous use containing at least 95% polyclonal IgGs (Kiovig, Sanquin). We will refer to this product as intravenous immunoglobulin (IVIg). In order to isolate anti-*S. aureus* specific IgGs from IVIg, a column with coupled bacteria was prepared. For this purpose, we chose the *S. aureus* strain Newman *ΔSpA ΔSbi*, which lacks both main IgG-binding proteins of *S. aureus*, to avoid non-specific retaining of antibodies. Bacteria were grown overnight in Mueller-Hinton Broth (MHB), and subsequently diluted in fresh MHB and grown to OD^660nm^ 0.5. Bacteria were resuspended from growth medium in PBS, fixed with 1% formaldehyde in PBS for 30 min at 4°C with gentle mixing and washed again in PBS. Finally, bacteria were resuspended to OD^660nm^ of 1.0 in coupling buffer (0.2 M NaCO_3_ and 0.5 M NaCl pH 8.0) and 7.5 mL recirculated over a 5 mL HiTrap NHS-column (GE healthcare). Column was washed alternating with 0.5 M ethanolamine in 0.5 M NaCl (pH 8.3) and 0.1 M acetate buffer in 0.5 M NaCl (pH 4.0) according to the manufacturer’s instructions. Based on OD^660nm^, 70% of the input was coated resulting in ± 43 x 10^9^ coupled bacteria.

For the isolation of affinity-purified IgGs, the column was loaded at 2 mL/min with 1 g IVIg at 10 mg/mL. Column was washed with PBS, eluted with 100 mM Glycine-HCL (pH 2.7) and peak fractions for OD^280nm^ directly neutralized with 1 M Tris (pH 8.0). IgG eluate was dialyzed overnight against PBS. Finally, only 1.15 mg affinity purified IgG was recovered.

### ELISA to Verify Enrichment of *S. aureus* Human IgGs From IVIg Preparation

To verify the enrichment of anti-*S. aureus* human IgG, the original and affinity purified IVIg were compared in an ELISA (detailed protocol in van den Berg et al., 2015). Briefly, logarithmic grown bacteria were coated at 5x106 per well (50 µl) in PBS overnight at 4°C in a high-binding microplate (Greiner). Plates were blocked with 4% BSA in PBS/Tween-20 (0.05%). Serial dilutions of IgG in PBS/Tween-20 with 1% BSA were added and bound IgG detected with F(ab’)2-goat-anti-Hu-IgG (Fc-gamma)-HRP labelled (Jackson) and TMB as substrate. Reaction was stopped with H_2_SO_4_ and the absorbance was measured at 450nm.

### Phagocytosis Assay

Phagocytosis was performed in phagocytosis buffer RPMI-HSA. In a round-bottom 96-well plate (Greiner), 20 µl of fluorescent bacteria (3.75 x 10^7^ CFU/mL) were mixed with 20 µl of a concentration range of human serum and/or purified antibodies for opsonization, for 15 min at 37°C on a plate thermoshaker (750 rpm). Subsequently, 10 µl of PMNs (7.5 x 10^6^ cells/mL) were added to reach a 10:1 bacteria-to-cell ratio in a final volume of 50 µl. The reaction was incubated for other 15 min at 37°C on a plate thermoshaker (750 rpm). Samples were then fixed for 30 min with 100 μL cold formaldehyde at a concentration of 1.5% (diluted from 10% aqueous methanol free, formaldehyde, Ultra pure; Polysciences, Inc.). Samples were either acquired immediately after 30 min of fixation at 4°C or after overnight fixation.

### Standard Data Acquisition of Phagocytosis by Flow Cytometry

Samples were acquired on a FACSVerse flow cytometer with Universal Loader (Becton Dickinson) equipped with a three-laser system (405, 488, 633 nm), 8-color (4-2-2) configuration and BD FACSuite software version 1.06.

In our standard acquisition set up, only neutrophil-related parameters were analyzed. Although we used density gradient centrifugation to isolate highly pure PMNs, gating of cells is still required to eliminate cell debris and possible free or clumped bacteria. Neutrophils were therefore gated adjusting Forward scatter (FSC) and Side scatter (SSC) parameters to linear scale. A threshold at FSC signal was also set, so that free bacteria were excluded from the acquisition. The fluorescence of FITC-, GFP-, or mAm-labelled staphylococci associated with neutrophils was acquired in logarithmic scale. A total of 7,000 events were collected for each sample gated on neutrophils. All data were exported as Flow Cytometry Standard format 3.0 files (FCS files).

### Analysis of Flow Cytometry Phagocytosis Data

**Figure 1:**
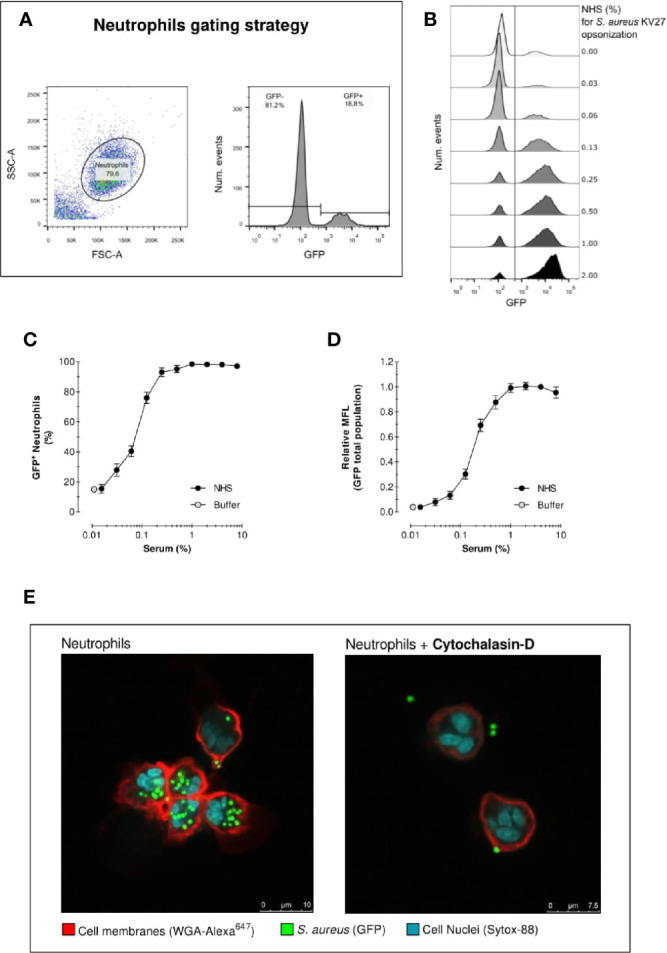
Acquisition of phagocytosis assay by flow cytometry. Acquisition of neutrophil phagocytosis of *S. aureus* KV27 opsonized with normal human serum (NHS) at a 10:1 bacteria-to-cell ratio after 15 min incubation. **(A)** Gating of PMNs in the linear FSC and SSC, required to eliminate cell debris and possible free or clumped bacteria from the analysis (left graph). Representative histogram describing the GFP intensity per number of events. The total population is composed by a percentage of GFP- and GFP+ PMNs (right graph). **(B)** Typical histogram overlay of the GFP intensity distribution of the total neutrophil population per each concentration point of opsonizing NHS. **(C)** Phagocytosis expressed as the % of GFP+ neutrophils per increasing % of NHS. Mean ± SEM for n=8–25 **(D)** Phagocytosis expressed as the Mean Fluorescence (MFL) of neutrophils relative to the MFL obtained with opsonization with 4% NHS. Mean ± SD for n=8–25. **(E)** Confocal image of *S. aureus* KV27 (green, GFP) incubated with 4% NHS engulfed by human neutrophils (red, membrane stain WGA-Alexa647 and blue, nuclear stain SYTO-82), control (right image) or treated with actin-blocking agent cytochalasin-D (left image).

FSC files were analyzed by FlowJo (Version 10; Treestar US, Ashland, OR). Our analysis of phagocytosis is based on the variation in the fluorescence intensity of neutrophils. This is easily visualized in histograms showing the number of cells distributed based on their fluorescence intensity (**Figures 1A, B**). The fluorescence intensity often results in two defined peaks: the GFP-negative cells that did not interact with fluorescent bacteria (left side), and of phagocytosing GFP-positive cells (right side). The main parameters of interest are: (1) the percentage of cells with a positive fluorescent signal (% positive cells), representing the part of the population that interacted with bacteria in our experimental conditions (2) the mean fluorescence (MFL) of all neutrophils. Because MFL values differ between experiments due to use of different batches of bacteria and labels, different Flow Cytometers with their respective instrument settings, and different donors, MFL data are preferably expressed relative to an optimal reference condition. To evaluate the different concentration curves in a single value, the Area Under the Curve (AUC) was calculated using GraphPad Prism (version 8). Therefore, serum concentration values were converted to their logarithmic number, and AUC was calculated using all serum concentrations with baseline correction for the % GFP+ Neutrophils of control samples (neutrophils and bacteria without serum).

### Alternative Data Acquisition of Phagocytosis (Neutrophils and Bacteria)

We adapted the flow cytometry protocol to measure the percentage of bacteria taken up by the neutrophils, as usually done in a classical phagocytosis assay. Samples pre- and post-phagocytosis were acquired on a FACSVerse flow cytometer. To acquire both bacteria (~1 µm diameter) and neutrophils (~12 µm), FSC and SSC parameters were set in logarithmic mode. To eliminate the substantial background events due to the full-scale log amplification for FSC and SSC, neutrophils were fluorescently labelled post-phagocytosis with 0.3 µg/ml LDS-751 nuclear stain (Thermofisher) for 5 min at 4°C without washing. GFP (*S. aureus*) and LDS-751 (PerCP channel setting) (neutrophils) signals were acquired on logarithmic scale and both used as threshold signals to collect only bacteria and neutrophils. A total of 50,000 events was acquired.

Setting quadrants in the GFP versus LDS-751 dot plot enables counting the number of free bacteria (GFP+/LDS-), neutrophils (GFP-/LDS+) and neutrophils containing bacteria (LDS+/GFP+). Hereby both cell types are acquired with minimal noise, enabling to calculate the ratio of bacteria (GFP+) versus neutrophils (LDS+). This setup allows monitoring of the percentage of neutrophils that participate in phagocytosis (LDS+/GFP+ fraction of LDS+ events), as well as the MFL value of the neutrophil population, but also the percentage of bacterial uptake reflected by the drop-in ratio, as alternative for actual concentration determinations (**Figure S3**).

### Microscopy Analysis of Phagocytosis

To analyse phagocytosis *via* light microscopy, samples were not fixed with formaldehyde, but immediately centrifugated onto glass slides using a cytospin-3 (Shandon), air dried and subsequently stained with the rapid Diff-Quick (Dade Behring) procedure. Pictures were taken with a Sony Nex-5 camera mounted without lens on an Olympus BX50 microscope.

For confocal microscopy, formaldehyde-fixed phagocytosis samples were mixed with Alexa Fluor^647^-labelled WGA (3 µg/ml; Molecular Probes) and SYTO-82 Orange Fluorescent Nucleic Acid Stain (5 µM; Molecular Probes) for 5 min before cytospin preparation. Air-dried cytospin slides were mounted with Poly-Mount and a cover slip. For confocal microscopy, samples were viewed on a Leica TCS SP5 using the 488 Argon laser line, 543 and 633 Helium-Neon laser line with a TD 488/561/633 dichroic beam splitter. Samples were viewed with two successive sequential scans to eliminate spill-over signals.

## Results

### Basic Parameters for the Acquisition of Neutrophils Phagocytosis of *S. aureus* by Flow Cytometry

Flow cytometry is a convenient high-throughput method for the evaluation of phagocytosis of fluorescently labelled *S. aureus* by neutrophils in suspension. In our standard assay set up we mix neutrophils with bacteria previously opsonized with pooled serum from healthy donors (normal human serum (NHS)), to enhance the engagement of cell surface receptors. By opsonizing bacteria with increasing concentrations of NHS, we can assess the impact of opsonization on phagocytosis.

As described in detail in the methods section, we evaluated the outcome of phagocytosis from cell-derived parameters. First, PMNs (containing >90% neutrophils) were gated to exclude cell debris and free bacteria from the analysis of internalized fluorescent *S. aureus*. **Figure 1A** shows an example of neutrophils gating, as well as the identification of the “GFP-” and “GFP+” cells in the GFP fluorescence histogram. The distinction between GFP negative and positive cells is defined by the autofluorescence signal of neutrophils in the absence of bacteria. **Figure 1B** shows the results in the form of overlaid histograms, representing the distribution of GFP fluorescence in the PMN population. With increasing serum concentrations, a marked increase of GFP-positive neutrophils is observed, together with the coherent increase of their fluorescence intensity, indicating that a larger number of cells was interacting with an increasing number of bacteria.

**Figure 1C** shows the percentage of GFP-positive neutrophils, thus the proportion of population that interacted with bacteria for each serum concentration. In the absence of serum, still 15% of PMNs interact with *S. aureus*. Conversely, when serum is introduced neutrophil engagement spikes rapidly. Already at a serum concentration of just 0.25%, more than >90% neutrophils are phagocytosing. Since isolated PMN fraction consists of ~95% neutrophils, 1–5% eosinophils (non-phagocytosing) and a few death cells, a non-phagocytosing fraction is always expected. We can reasonably conclude that >90% positive values indicate that virtually all neutrophils interacted with bacteria.

**Figure 1D** represents the mean fluorescence (MFL) of the neutrophil population, which summarizes the overall phagocytic activity of neutrophils considering both GFP- and GFP+ cells. This graph shows that, despite the percentage of neutrophils involved in phagocytosis reached a plateau after 0.25% opsonization, higher serum concentrations stimulate the cells to continue engulfing more bacteria.

One limitation of standard flow cytometry analysis is the inability to distinguish between bound or internalized bacteria within the GFP+ neutrophils. **Figure 1E** shows two representative confocal images of a phagocytosis experiment. The picture on the left clearly indicates that all bacteria are inside when opsonized with 4% NHS. The picture on the right shows a phagocytosis assay with neutrophils treated with actin-blocking agent Cytochalasin-D, which impairs phagocytosis. The confocal images thus demonstrate that despite the inability of flow cytometry to distinguish non-internalized bacteria, the number of bacteria attached to the cell surface is negligible, thus the results of flow cytometric analysis are attributable to the internalized bacteria. In addition, phagocytosis was performed on ice (with shaking) in parallel with 37°C, both conditions first opsonized for 15 min at 37°C. Keeping the conditions strictly cold, only background association was observed comparable with no serum (**Figure S2**).

Together our results confirm that, under these assay conditions, opsonization of bacteria with serum is essential for successful phagocytosis by neutrophils.

### Incubation Time and Bacteria-To-Cell Ratio Dictate the Dynamics of the Phagocytosis Assay

The dynamics of phagocytosis *in vitro* depend on assay parameters that regulate the contact between cells and bacteria. Incubation time and bacteria-to-cell ratio are two main parameters that govern and influence the contact phase. In our standard phagocytosis assay, opsonized bacteria and cells are incubated together with vigorous shaking for 15 min at a 10:1 bacteria-to-cell ratio. To justify these choices, experiments are shown to evaluate the effect of varying the incubation time and ratio on the outcome of the phagocytosis assay.

**Figure 2:**
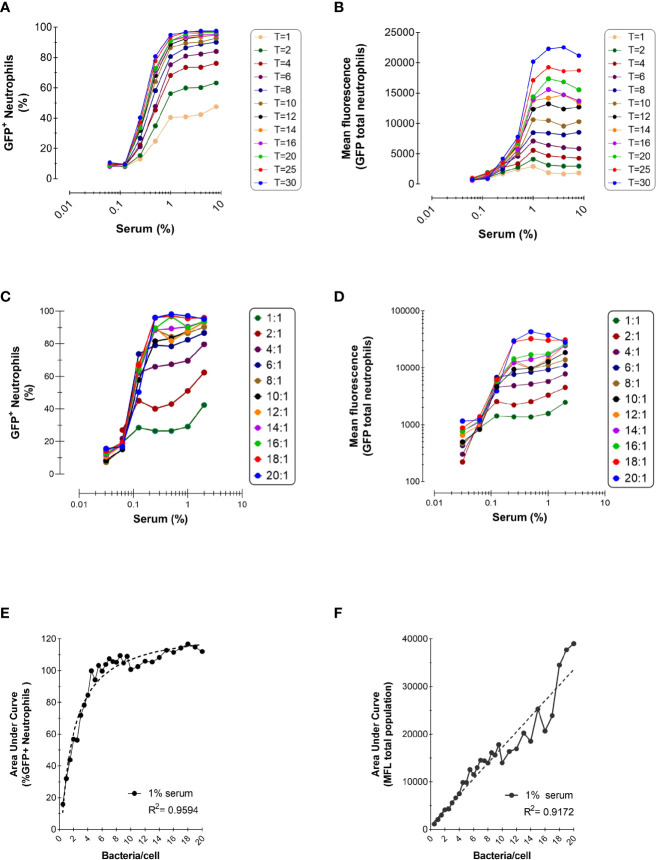
Effect of Incubation Time and Bacteria-to-Cells Ratio on Opsonophagocytosis. **(A, B)** Time-dependent phagocytosis of KV27-GFP in the presence of pooled NHS. Bacteria and PMNs at a 10:1 ratio were incubated at 37°C in tubes and samples were withdrawn for each time point into ice cold formaldehyde to stop the reaction. Data are expressed as % GFP+ PMNs **(A)** and Mean fluorescence **(B)**. Representative experiment of n=2. **(C, D)** Phagocytosis of GFP-expressing Newman *ΔSpA ΔSbi* using different ratios bacteria to neutrophils. Phagocytosis of NHS opsonized bacteria expressed as % GFP+ neutrophils for depicted ratios **(C)** and as MFL of total neutrophils **(D)**. **(E, F)** For 1% serum at different ratios, data of % GFP+ neutrophils are fitted to a nonlinear regression curve [least squares fit; GraphPad, Agonist vs. response (three parameters)] **(E)** and data of total MFL are fitted to a linear regression curve **(F)**. Data pooled from n=2 experiments.

To investigate how incubation time affects phagocytosis efficiency, we performed the same phagocytosis assay described in the previous section, with GFP-expressing *S. aureus* KV27 opsonized with increasing concentrations of NHS, but we stopped the incubation with cells between 1 to 30 min. **Figure 2A** shows that, as incubation time increases, so does the percentage of phagocytosing neutrophils, demonstrating that a longer incubation time increases the probability of a cell to encounter a bacterium. It is also clear that phagocytosis is a fast process; after only 1 min, more than 40% neutrophils phagocytosed bacteria, and by 8 min more than 90% neutrophils participated.

For all incubation times, we confirmed the observation from the previous section that a higher opsonization of bacteria enhanced phagocytosis. However, when bacteria were treated with 1% or more serum phagocytosis reached a plateau, suggesting that bacteria were saturated by opsonins and neutrophil stimulation reached its maximum.

**Figure 2B** represents the total mean fluorescence values, reflecting the mean number of phagocytosed bacteria per neutrophil. In accordance with **Figure 2A**, the MFL increased proportionally with incubation time, indicating that neutrophils engulf more bacteria when offered more time. When all neutrophils participated in the phagocytosis process, reaching their plateau after 8 min (**Figure 2A**), neutrophils kept engulfing more bacteria per cell the more the incubation time was extended (**Figure 2B**).

Next, we evaluated the outcome of phagocytosis when varying the bacteria-to-cell ratio. Low bacteria-to-cell ratios, from 0.5:1 to 20:1, were chosen to emulate the physiological ratios at the site of infection. **Figures 2C, D** show that both the percentage of positive neutrophils and total mean fluorescence are still dependent on the serum concentration for all ratios tested, and the percentage of neutrophil population involved reaches a plateau at ratio 8:1.

To better compare the serum-concentration curves for each ratio, the area under the curve (AUC) was calculated for both the percentage of positive neutrophils and their total MFL, as representative number for the phagocytic capacity of neutrophils for all serum concentrations. As already evident from **Figure 2C** (depicting a selected part of all ratios tested), with increasing ratios the AUC for neutrophils participating in phagocytosis reaches a plateau at a ratio of 6:1 (**Figure 2E**). Clearly, **Figure 2F** shows that, with increasing ratios, the ability to phagocytose more bacteria per neutrophil accumulates and under these conditions, does not reach a maximal plateau of phagocytic capacity.

### Correlation of Microscopy and Flow Cytometry Analysis

A phagocytosis assay is typically evaluated by measuring engulfed bacteria inside cells, either by microscopy or flow cytometry. Here we compared the automated and more throughput flow cytometric analysis to the classical microscopic counting, to ensure that a higher MFL signal indeed reflects phagocytosis of more bacteria per neutrophil.

To provide two different phagocytosis conditions, bacteria were opsonized with either NHS or heat-inactivated serum (HI-NHS), in which key complement proteins are inactive. Due to the absence of complement activation, we expect a marked decrease in the efficiency of phagocytosis, since engulfment will be mainly mediated by the interaction of Fc receptors (FcRs) with opsonizing IgGs and a few IgAs. This will therefore give us the opportunity to compare two markedly different results with both techniques.

**Figure 3:**
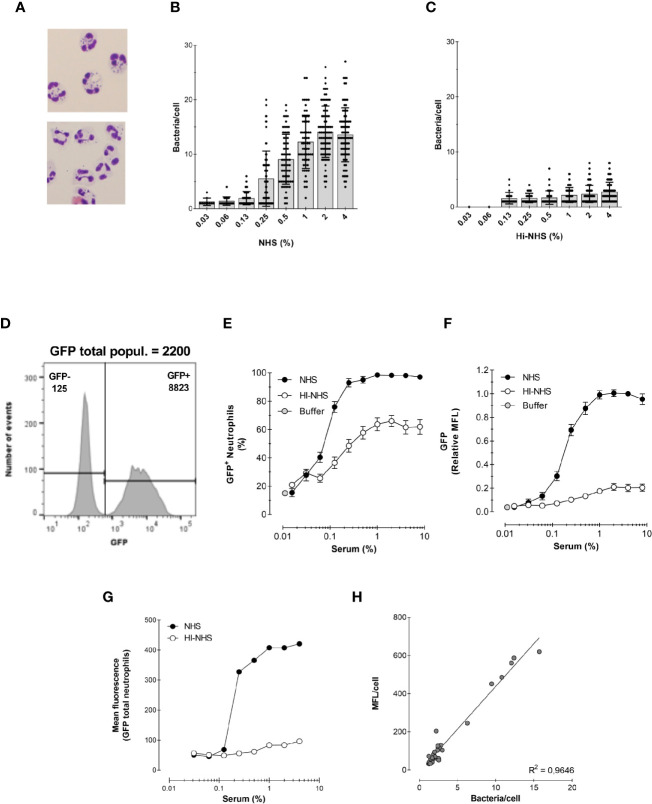
Correlation between microscopy and flow cytometry. **(A)** Example of cytospin preparation after phagocytosis for 15 min with 1% NHS (top) and 1% HI-NHS (bottom). **(B, C)** Counting bacteria per neutrophil on cytospin preparations after phagocytosis with NHS **(B)** and HI-NHS **(C)**. Individual counts and means ± SD (n=25–100) for only neutrophils with bacteria. **(D)** Representative histogram to show GFP− and GFP+ population and their corresponding MFL value versus their total MFL. **(E, F)** Flow cytometry analysis of phagocytosis with NHS and HI-NHS shown as % GFP+ neutrophils **(E)** and relative MFL to 8% NHS **(F)** for total neutrophil population. **(G, H)** Representative figure for MFL of only the GFP+ neutrophil population **(G)** and correlation of that with microscopic counts per cell **(H)** for n=2 experiments.

**Figures 3A–C** show the analysis of the assay *via* microscopy, where engulfment is typically evaluated by counting visually the number of bacteria per cell. The average number of bacteria per 50–100 cells is reported in **Figures 3B, C**. Phagocytosis is heterogeneous throughout the neutrophil population, as already evidenced in the relative broad fluorescence histograms shown in **Figure 1B**. As expected, the average number of bacteria per cell was higher with increasing concentrations of serum. In addition, a marked decrease in phagocytosis efficiency was observed when bacteria were opsonized with HI-NHS (**Figure 3C**), when compared with NHS (**Figure 3B**), showing that complement greatly contributes to enhance the phagocytic process.

**Figures 3D–F** show the analysis of the same experiments by flow cytometry. In contrast to the laborious microscopic analysis, the flow cytometer automatically evaluates the fluorescence of ~7000 neutrophils in a short time (exemplified in **Figure 3D**). This way of analysing the experiments clearly shows the presence of a significant portion of non-phagocytosing cells, which was also clear by microscopy analysis. However, for the evaluation by microscopy, only neutrophils containing bacteria were analyzed. The results of the experiments are presented in the standard format (explained in *Section 1*) as % of phagocytosing GFP+ neutrophils (**Figure 3E**) and relative GFP mean fluorescence of the total neutrophil population (**Figure 3F**).

To be able to compare the counted numbers with a representing fluorescence value, the mean fluorescence of the GFP+ neutrophil population alone was determined **(** **Figures 3D, G**). Plotting the bacteria/cell (microscopy) versus MFL/cell (flow cytometry) showed a linear regression with an R^2^ = 0.96, indicating that the flow cytometric MFL reflects number of phagocytosed bacteria (**Figure 3H**).

### Dissecting the Role of IgG, IgM, and Complement in Phagocytosis of *S. aureus*

In the previous section we showed that inactivating complement deposition on *S. aureus* severely impairs bacterial uptake. In this section we will elaborate more on the single contributions of serum opsonins in the phagocytosis of *S. aureus*.

**Figure 4:**
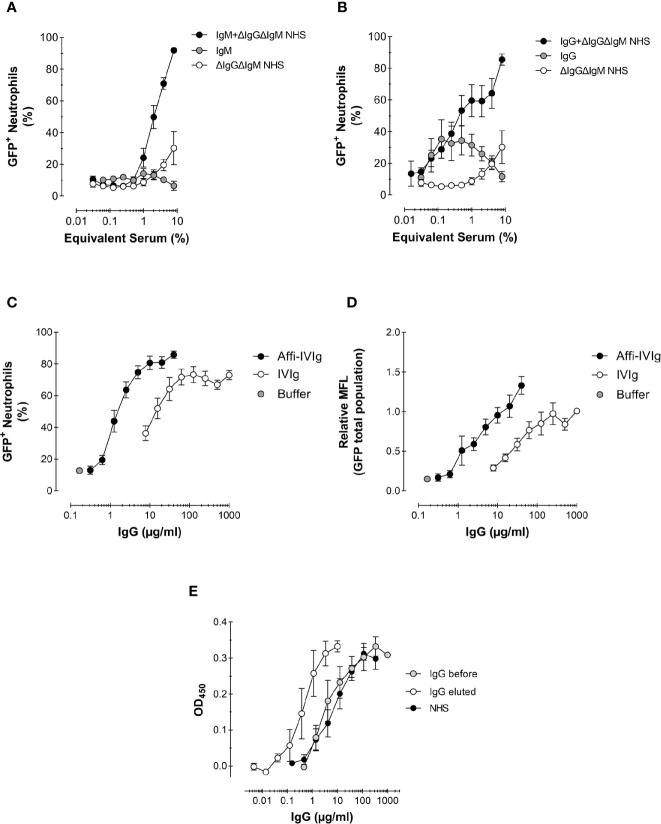
Role of antibodies and complement in the phagocytosis of *S. aureus.* **(A, B)** Phagocytosis expressed as percentage of GFP+ neutrophils of Newman *ΔSpA ΔSbi* opsonized with a complement source depleted of antibodies (ΔIgGΔIgM NHS), NHS-purified IgM **(A)** or IgG **(B)**, or the combination of IgM **(A)** or IgG **(B)** reconstituted with complement. For purified IgM the equivalent concentration present in 8% serum is used, 120 µg/ml **(A)**, and for purified IgG that is 1,000 µg/ml **(B)**; these concentrations were also used to reconstitute into 8% ΔIgGΔIgM NHS. Data represent Mean ± SEM for n=2–8. **(C, D)** Phagocytosis of *S. aureus* KV27 opsonized with either commercial IgGs for intravenous use (IVIg), or the same IgGs affinity purified on a *S. aureus* Newman *ΔSpA ΔSbi* column (Affi-IVIg). Phagocytosis is expressed as the % of GFP+ neutrophils per increasing percentage of IVIgs or Affi-IVIgs. Mean ± SEM for n=7 **(C)** and as the Mean Fluorescence (MFL) of the total neutrophils engulfing GFP-expressing bacteria **(D)**. Mean ± SEM for n=6. **(E)** Binding specificity of NHS, IVIg (IgG total) and Affi-IVIg (IgG eluted) antibodies assessed by ELISA, using *S. aureus* strain Newman *ΔSpA ΔSbi.* Mean ± SD for n = 2.

First, we analyzed the contribution of complement proteins to opsonization. To obtain an antibody-free complement source (denoted as ΔIgGΔIgM NHS), we depleted pooled serum from all IgG and IgM antibodies. When bacteria were opsonized with ΔIgGΔIgM NHS, phagocytosis efficiency was scarce (**Figures 4A, B**). Only at the highest concentrations of 8 and 4% ΔIgGΔIgM NHS used, a small proportion of neutrophils were involved in phagocytosis. This result suggests that at lower concentrations of serum, antibodies are required to kick start complement deposition *via* the classical pathway.

Subsequently, we analyzed the phagocytic stimulus offered by monomeric IgGs and multimeric IgMs antibodies, which were recovered during the depletion of pooled serum. We used the equivalent concentrations of purified IgM and IgG present in 8% serum as 120 µg/ml for IgM (~1.5 mg/ml in serum) and 1,000 µg/ml for IgG (~12.5 mg/ml in serum) in Ig conditions only as well as reconstituted into ΔIgGΔIgM NHS. Additionally, we evaluated commercial IgGs for intravenous use (IVIg), which are usually offered to patients in need for a supplement of antibodies. As expected, purified IgMs alone did not stimulate any phagocytosis (**Figure 4A**). On the contrary, purified IgGs triggered phagocytosis, but reached a plateau of 40% cells involvement by ~60 µg/mL (shown as ~0.6% equivalent serum), suggesting a saturation of the bacterium and/or maximal engagement of FcγRs of neutrophils (**Figure 4B**). We also compared the phagocytic efficiency of the IgM or IgG antibody class from pooled serum recombined with their original complement source ΔIgGΔIgM NHS. In both cases, the percentage of phagocytosing neutrophils triplicated. We observed that when complement was present, IgM was slightly more efficient then IgG in stimulating phagocytosis (compare **Figure 4A** with **Figure 4B**). In fact, oligomeric IgM are strong complement activators, providing a preferential docking station for C1q deposition (37).

Also increasing concentrations of IVIg were used to opsonize bacteria, resulting in >70% phagocytosing neutrophils (**Figures 4C, D**). The highest phagocytosis level was reached at IVIg concentrations of 200 µg/mL. This result confirms that commercial preparations also contain sufficient anti-Staphylococcal antibodies to efficiently stimulate *S. aureus* phagocytosis.

To show more directly the presence and capacity of specific anti-*S. aureus* antibodies within this pool of IgG, we enriched the IVIg solution by passing it over a column with sepharose-coupled formalin-fixed *S. aureus* Newman *ΔSpA ΔSbi* (Affi-IVIg). This strain was purposely chosen to prevent the aspecific capturing of antibodies by the IgG-binding proteins on the bacterial surface. The *S. aureus-*bound IgGs were then recovered and re-tested in the phagocytosis assay. To reach the same level of phagocytosis of the original IVIg, a 10-fold lower concentration of enriched IgGs was sufficient, in concordance with the 10-fold enrichment of anti-Staphylococcal antibodies measured by ELISA with coated bacteria (**Figure 4E**). The enrichment overall enhanced the percentage of phagocytosing neutrophils (**Figure 4C**), as well as the uptake of fluorescent bacteria at maximal concentrations used (**Figure 4D**).

In summary, purified IgGs provide a better phagocytic stimulus than IgM, but in combination with complement, their phagocytosis efficiency is comparable. We also observed that by enriching IgG preparations with *S. aureus*-specific antibodies, we can enhance phagocytosis and potentially aid the clearance of the bacterium in patients with lower amounts of specific antibodies.

### Phagocytosis Efficiency of Single Serum Does Not Correlate With Its Antibody-Dependent Phagocytosis or Its Complement Activity

We observed in the previous section that pooled human serum, which is rich in anti-Staphylococcal antibodies, makes an excellent opsonic source for *S. aureus* phagocytosis. However, the humoral response differs in every individual, as well as complement activity. In this section we assess the ability of 24 sera from healthy donors to mediate phagocytosis of *S. aureus* strain Newman *ΔSpA ΔSbi* and we compare it to pooled serum (NHS).

**Figure 5:**
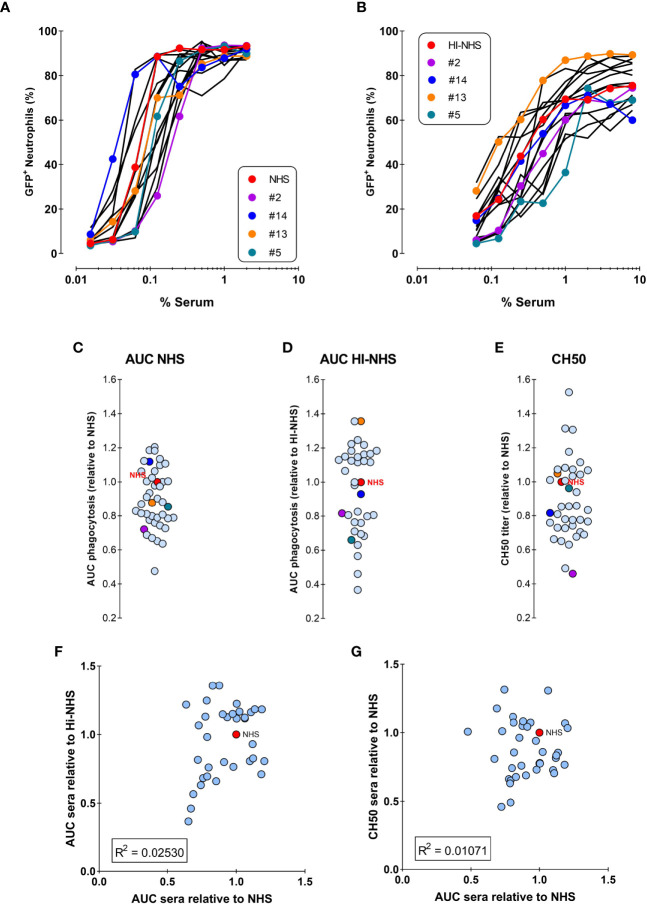
No correlation between phagocytosis by NHS versus HI-NHS or complement activity. **(A, B)** Phagocytosis of individual sera as compared to the same sera present in the pooled NHS expressed as % GFP+ neutrophils for NHS **(A)** and Heat-Inactivated serum (HI-NHS), without complement activity **(B)**. Representative experiment for 16 individual sera and their pool (NHS) with some color coded for comparison. **C–E**) AUC value for each individual serum curve expressed relative to NHS phagocytosis (**C**; n=40****), HI-NHS phagocytosis (**D**; n=37****), and CH50 value relative to NHS (**E**; n=37****). **(F, G)** No correlation between phagocytosis AUC for NHS versus HI-NHS **(F)** or for phagocytosis versus CH50 for NHS **(G)** with n=37.

**Figure 5A** shows the opsonic capacity of individual sera and their pooled NHS represented as the percentage of engulfing neutrophils. All sera succeeded in mediating phagocytosis, although they displayed some heterogeneity. **Figure 5B** represents the percentage of phagocytosis induced by the same sera after heat-inactivation, to eliminate complement activity. The same data were represented in **Figures 5C, D** where the areas under the curve (AUC) were related to NHS. We observed that the hierarchy observed in **Figure 5A** is not maintained in **Figure 5B**, exemplified by the colored individual sera. This suggests that the antibody repertoire mediating phagocytosis *via* FcγR is not the only determinant factor responsible for the final strength of the serum. In fact, no correlation was found between the potency of normal and heat inactivated sera (**Figure 5F**).

We then investigated whether complement could have a predominant role in the final efficacy of a serum in mediating *S. aureus* phagocytosis. To assess the complement activity of each serum independently from its antibody content, we performed a CH50 assay, which defines the percentage of serum that lyses 50% of sheep erythrocytes pre-sensitized by specific antibodies. When compared to NHS complement activity as a reference, sera still displayed heterogeneity (**Figure 5E**), however they also did not show any correlation with the potency of the related full sera (**Figure 5G**).

**Figure 6:**
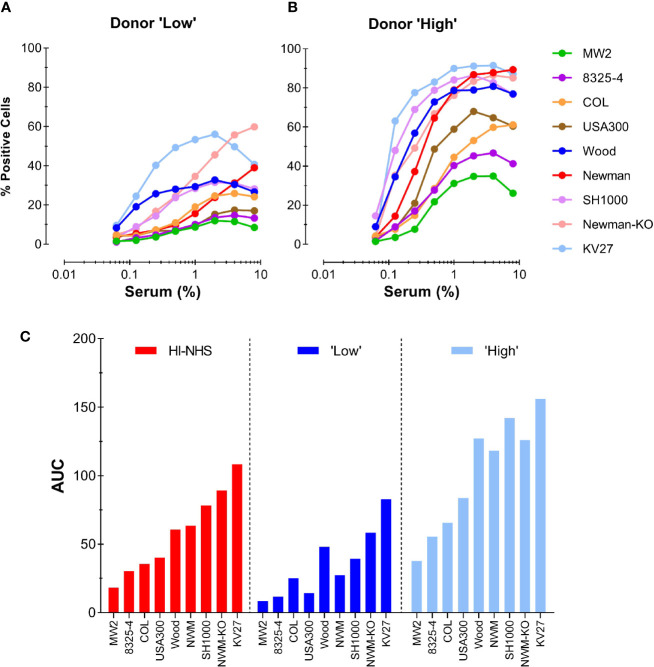
Consistent donor opsonic capacity for multiple *S. aureus* strains. **(A, B)** Phagocytosis of different *S. aureus* strains opsonized with HI-serum of two individual donors chosen from the panel of 37 individual donors. Donor with “Low” **(A)** and donor with “High” **(B)** opsonic capacity as determined for Newman *ΔSpA ΔSbi* were compared with HI-NHS (graph not shown). **(C)** AUC values for each individual *S. aureus* curve were ranked in ascending order for HI-NHS as opsonic source. Data are from one experiment using standard 10:1 bacteria to neutrophil ratio and 15 min phagocytosis time and expressed as % GFP+ neutrophils.

To further evaluate the competence of an individual donor serum for different *S. aureus* strains, we chose a “Low” (#15) and a “High” (#13) donor from the representative experiment shown in **Figure 5B**. These two donors were compared with the HI-NHS pool in opsonophagocytosis of 9 different *S. aureus* strains, including the KV27 and Newman *ΔSpA ΔSbi* strain as well as wild-type Newman. **Figures 6A, B** shows the phagocytosis curves for the different *S. aureus* strains opsonized by the “Low” and the “High” donor. There is a high variability among the tested *S. aureus* strains to be phagocytosed. Conversion of the data to AUC and ranking the different strains for HI-NHS in ascending order (**Figure 6C**) clearly shows that the opsonic capacity of a serum is universal for different *S. aureus* strains, indicative for common epitope recognition.

In conclusion, the potency of a single donor serum to opsonize *S. aureus* for efficient phagocytosis by neutrophils is not solely determined by its anti-staphylococcal IgG or intrinsic complement activity, but is comparable for different strains.

### Conditions to Clear All Bacteria Efficiently

**Figure 7:**
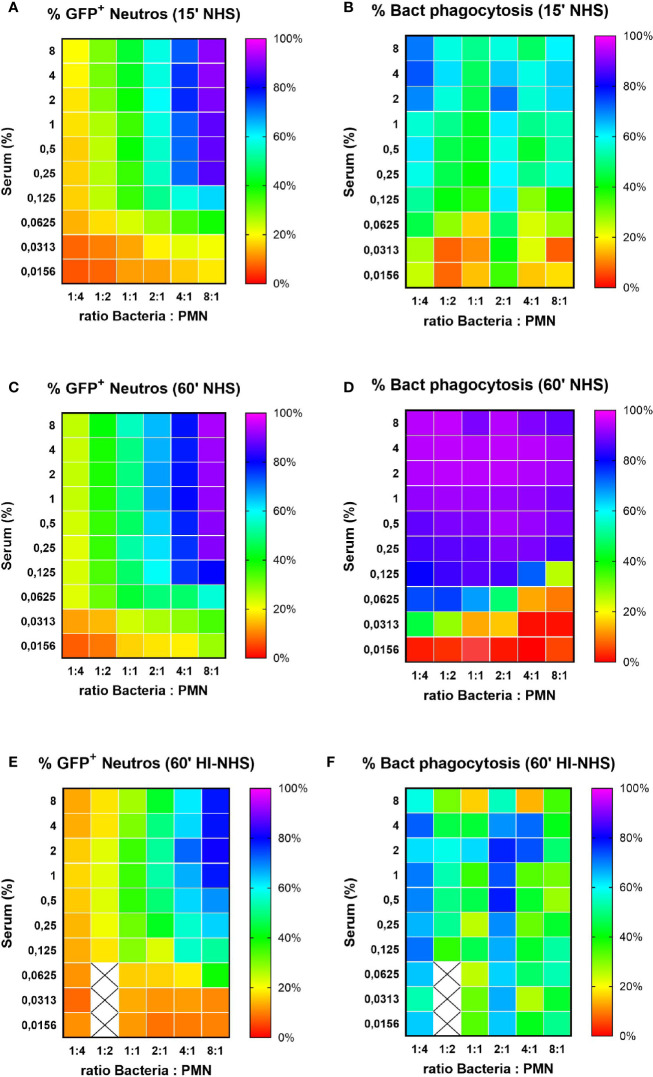
Percentage of phagocytosing neutrophils vs. % phagocytosed bacteria. Heat map visualization of both % GFP+ neutrophils (left side panels) and % bacterial phagocytosis (right side panels) for increasing ratios of bacteria to neutrophils. Results for 15 **(A, B)** and 60 **(C, D)** min phagocytosis in the presence of increasing concentration NHS and for 60 min phagocytosis in the presence of HI-NHS **(E, F)**. Data are mean values of n=3 experiments, and X are missing data points.

Our standard phagocytosis assay provides efficient basic parameters to compare opsonization conditions, or factors that either inhibit or stimulate neutrophil phagocytic capacity. The former is exemplified by e.g. *S. aureus* immune evasion proteins that interfere with C3b deposition on bacteria (38) or IgG interaction with FcγRs (39). Modification of the assay also provides the opportunity to use flow cytometry to evaluate actual bacterial uptake; the percentage of phagocytosed bacteria. Using two fluorescent tags for neutrophils and bacteria during acquisition, both partners of the phagocytosis assay can be analyzed simultaneously. Phagocytosis was performed with a full serum opsonization range at different bacteria-to-cell ratios for both 15 and 60 min incubation time. The gating analysis strategy is depicted in **Figure S3** for samples with a starting ratio of 1:1 (**Figures S3A, B**) and 8:1 (**Figures S3 D, E**), in the presence of 4% NHS with immediate fixation. As phagocytosis proceeds with increasing serum concentrations and time, the percentage of free bacteria will decrease, which can be calculated from a decrease in bacteria-to-cell ratio, as compared to the starting situation. Representative example is shown in **Figure S3** for phagocytosis after 15 min in the presence of 4% NHS at a 1:1 (**Figure S3C**) and a 8:1 (**Figure S3F**) ratio. The multiple analysis of these data is depicted in heat-maps presented as percentage of GFP+ neutrophils (representing the % of all neutrophils that contain bacteria; *Section 2.11*) and percentage of bacterial phagocytosis (representing the % of all bacteria present that are taken up by the neutrophils; *Section 2.12*) (**Figure 7**). It is clear that at very low bacteria to neutrophil ratios (≤1:1), the percentage GFP+ cells remain low (**Figures 7A, C**; 15 min versus 60 min with NHS), but prolonging the incubation time enables efficient uptake of all available bacteria (**Figures 7B, D** **)**. For the higher bacteria to neutrophil ratios (≥2:1), the percentage of GFP+ neutrophils is much higher conform our previous data in **Figure 2**, but uptake of bacteria requires more opsonisation (**Figures 7A, B** for 15 min and **C, D** for 60 min). For all ratios, a prolonged incubation time is required to clear most of the bacteria from the incubation mixture. The same condition for heat-inactivated serum after 60 min incubation (**Figures 7E, F** **)** again stresses the requirement for active complement for proper opsonization to provide optimal uptake of all available bacteria.

### General Conclusion

Our flow cytometric method for the determination of neutrophil mediated phagocytosis of *S. aureus* especially pinpoints to the role of different opsonins that steer the uptake. Basic characteristics of the method were evaluated that in general influence the dynamics of the assay, and maybe adapted for different research questions. The comparison of normal serum versus heat-inactivated serum (30 min at 56°C) is an easy way to study the contribution of the complement system in the opsonophagocytosis process. We also compared that with purified IgG, IgM and human serum depleted for naturally present IgG and IgM as an alternative complement source. Changing opsonisation conditions affects the phagocytosis capacity of neutrophils, not always reflected in the percentage of neutrophils that engulf bacteria, but usually more clear in the number of ingested bacteria (reflected by the total mean fluorescence value). This was verified by microscopic counting. In general, individual healthy donors possess sufficient opsonic capacity for proper phagocytosis of different *S. aureus* strains, but with variability that is consistent for the tested strains. Finally, we showed that under proper conditions of prolonged time and around a 1 to 1 bacteria to neutrophil ratio, almost all bacteria present can be engulfed. Although several well described flow cytometric assays were already known, this method adds some new considerations in comparing different opsonisation conditions.

## Data Availability Statement

The raw data supporting the conclusions of this article will be made available by the authors, without undue reservation.

## Ethics Statement

The studies involving human participants were reviewed and approved by Blood was obtained from healthy donors after informed consent was obtained from all subjects, in accordance with the Declaration of Helsinki. Approval from the Medical Ethics Committee of the University Medical Center Utrecht was obtained (METC protocol 07-125/C approved on March 1, 2010). The patients/participants provided their written informed consent to participate in this study.

## Author Contributions

EB, IB, TJ, EV, and KV performed the experiments. EB, SR, and KV analyzed the data. EB, SR, and KK wrote the paper. JV, SR, and KV designed the research study and critically read the manuscript and provided constructive comments. All authors contributed to the article and approved the submitted version.

## Funding

This work was supported by the European Union’s Horizon 2020 research: H2020-MSCA-ITN (No. 675106 coordinated by Dr. Fabio Bagnoli, GSK Vaccines S.r.l., Siena, Italy) and ERC Starting grant (#639209, to SR).

## Conflict of Interest

EB is participating in a post-graduate studentship program at GSK.

The remaining authors declare that the research was conducted in the absence of any commercial or financial relationships that could be construed as a potential conflict of interest.

## Supplementary Material

Supplementary Figure 1 Chemical surface labelling with FITC do not impact opsonization and phagocytosis. *S. aureus* Newman *ΔSpA ΔSbi* expressing the violet excitable yellow fluorescent protein mAmetrine was additionally externally labeled with fluorescein isothiocyanate (FITC) and compared with the mock treated bacteria in the phagocytosis assay with NHS and HI-NHS. Phagocytosis was measured as % of mAm+ PMNs **(A, B)** and mAmetrine MFL of the total population **(C, D)**. **(E, F)** Control graphs showing that FITC MFL of PMNs only increases when bacteria are FITC-labeled. Representative experiment.

Supplementary Figure 2 On ice no phagocytosis occurs. *S. aureus* was opsonized with NHS at 37°C for 15 min, cooled on ice or kept on room temp for 10 min, and subsequently mixed with neutrophils (cold versus room temp) for a 15 min phagocytosis on ice or at 37°C. Phagocytosis is expressed as % GFP+ neutrophils **(A)** and GFP relative MFL (4% serum) of the total population **(B)**. Data are the mean of 2 experiments ± SD.

Supplementary Figure 3 Gating strategies for the alternative phagocytosis assay using FITC (or GFP) and LDS-753 (PerCP signal) as thresholod parameters for data aquisition. Shown are representative dot plots for a phagocytosis assays performed with putative bacteria to cell ratio 1:1 (upper panel) and 8:1 (lower panel). During the acquisition, bacteria to cell ratio was verified by gating both bacteria and PMNs by setting logarithmic scatter parameters (panel **A** for 1:1 and D for 8:1) or fluorescence parameters (panel **B** for 1:1 and **E** for 8:1). The actual observed ratio for the 1:1 appeared to be 1.1:1, and for the 8:1 ratio appeared to be 6.3:1, both in agreement for the scatter and the fluorescence based parameters. Dot plots are from the control samples before starting the experiments with 4% NHS, mixed on ice and immediately fixed with paraformaldehyde. Panels **C, F** are representative fluorescence dotplots for the 15 min phagocytosis timepoint with 4% NHS showing for the 1.1:1 ratio **(C)** 53.8% GFP+ neutrophils at a 0.45:1 ratio resulting in 59.8% bacterial phagocytosis, and for the 6.3:1 ratio **(F)** 96.6% GFP+ neutrophils and a 58.2% bacterial phagocytosis.

## Abbreviations

AUC, Area Under Curve; CH50, Classical Pathway Hemolytic Assay; CR, Complement Receptor; EC50, Half Maximal Effective Concentration; *FcRs, Fc receptors;* FITC, Fluorescein Isothiocyanate; FSC, Forward Scatter; GFP, Green Fluorescent Protein; HI-NHS, Heat-inactivated NHS; HSA, Human serum albumin; IgA, Immunoglobulin A; IgG, Immunoglobulin G; IgM, Immunoglobulin M; IVIg, Intravenous Immunoglobulin; PBS, Phosphate Buffer Saline; PMNs, *Polymorphonuclear* Leukocytes; LDS, Laser Dye Styryl; mAm, mAmetrine; MFL, Mean Fluorescence; MHB, Mueller-Hinton Broth; NHS, Normal Human Serum; RPMI, Roswell Park Memorial Institute Medium; Sbi, Second Immunoglobulin-Binding Protein; sGFP, Superfolder Green Fluorescent Protein; ShRBC, Sheep Red Blood Cells; SpA, Staphylococcal Protein A; SSC, Side Scatter; ΔIgGΔIgM serum, IgG and IgM-depleted Serum; THA, Todd Hewitt Agar; THB, Todd Hewitt Broth; TMB, 3,3’,5,5’-Tetramethylbenzidine; WGA, Wheat Germ Agglutinin.
