# Supplementary figures and images for: Use of Flow Cytometry to Evaluate Phagocytosis of Staphylococcus aureus by Human Neutrophils
Source: Front Immunol. 2021 Feb 19;12:635825. doi: 10.3389/fimmu.2021.635825 (PMC7934835; doi:10.3389/fimmu.2021.635825)

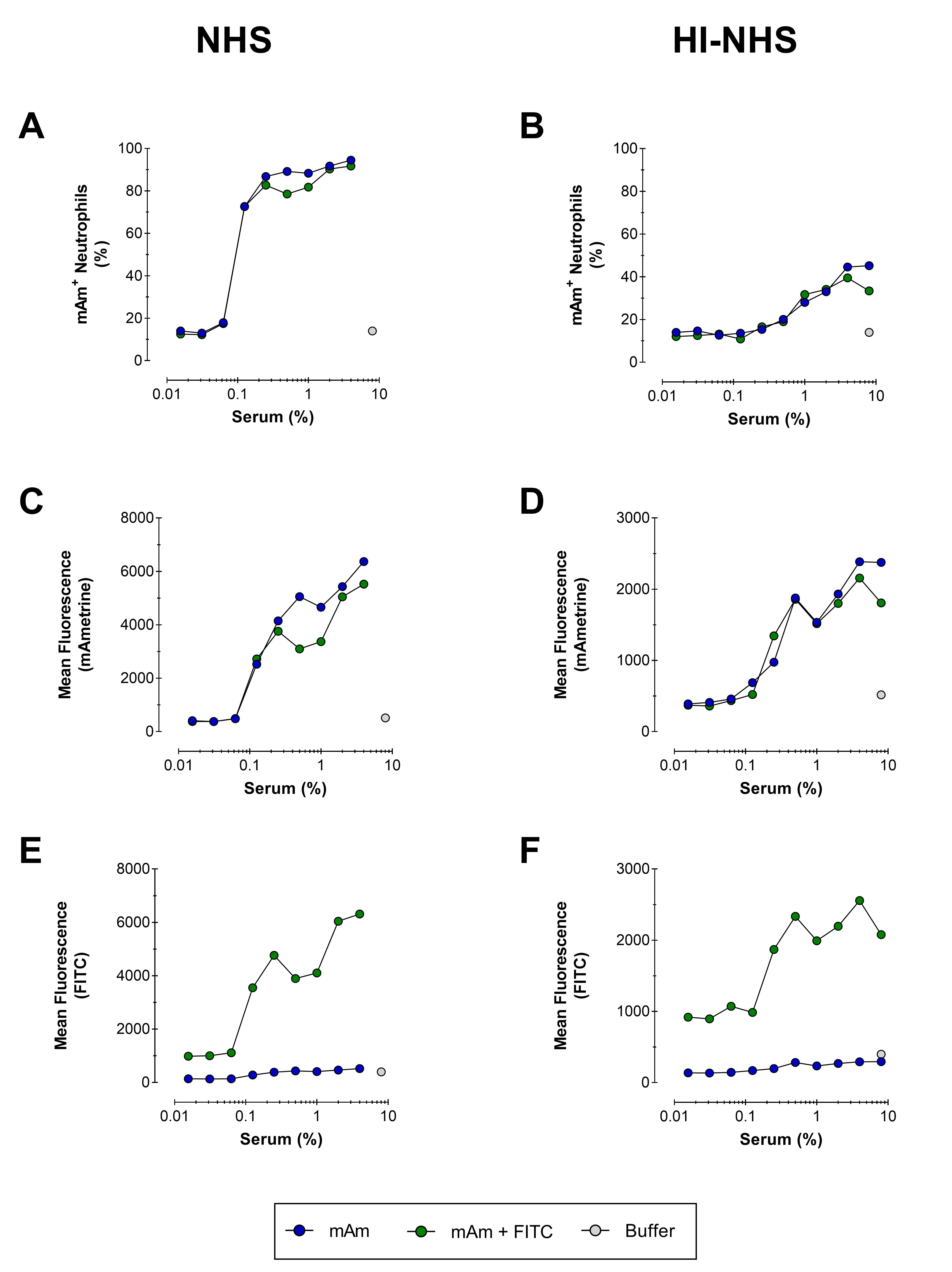

Supplement: Supplementary Figure 1 — Chemical surface labelling with FITC do not impact opsonization and phagocytosis. S. aureus Newman ΔSpA ΔSbi expressing the violet excitable yellow fluorescent protein mAmetrine was additionally externally labeled with fluorescein isothiocyanate (FITC) and compared with the mock treated bacteria in the phagocytosis assay with NHS and HI-NHS. Phagocytosis was measured as % of mAm+ PMNs (A, B) and mAmetrine MFL of the total population (C, D). (E, F) Control graphs showing that FITC MFL of PMNs only increases when bacteria are FITC-labeled. Representative experiment. [file Image_1.jpeg]

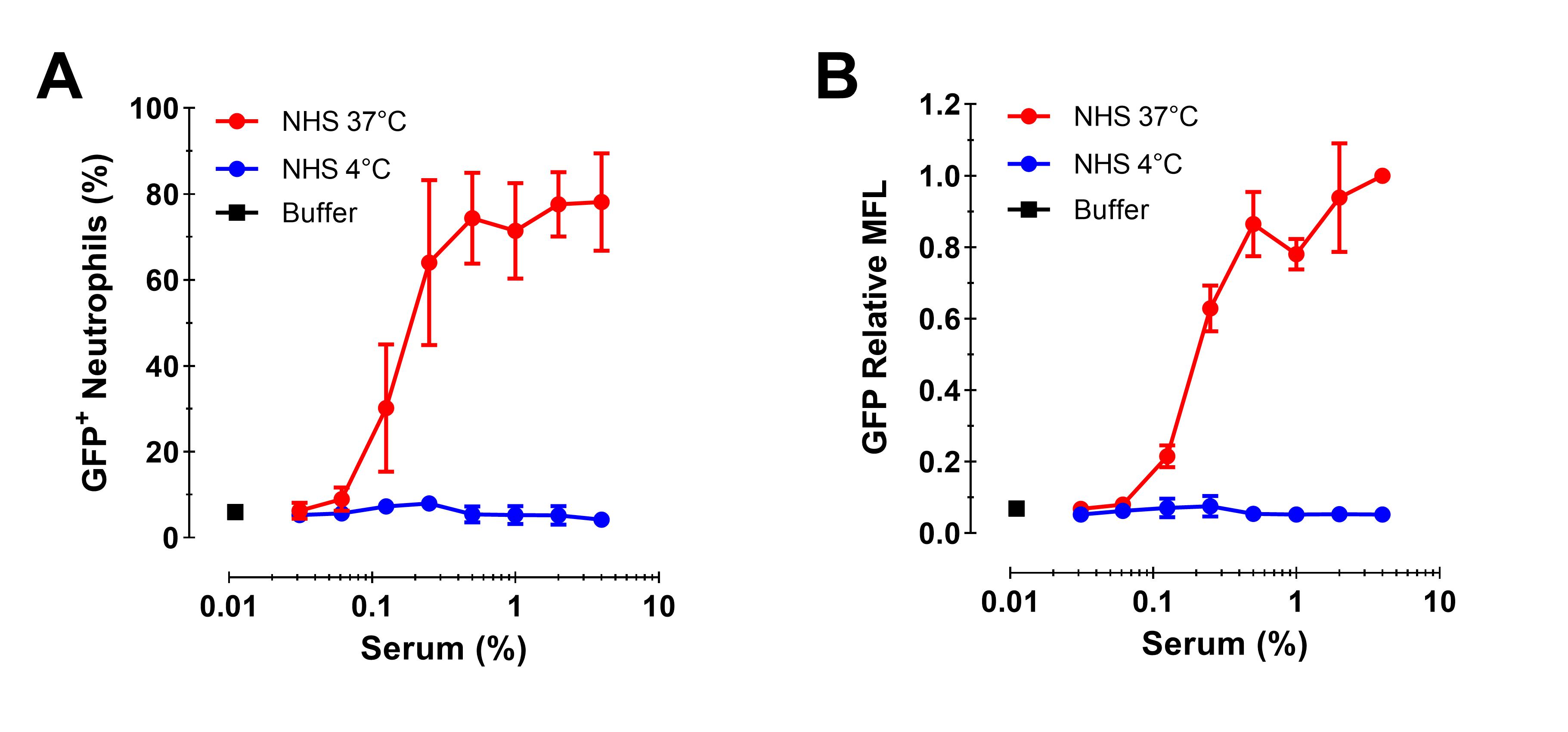

Supplement: Supplementary Figure 2 — On ice no phagocytosis occurs. S. aureus was opsonized with NHS at 37°C for 15 min, cooled on ice or kept on room temp for 10 min, and subsequently mixed with neutrophils (cold versus room temp) for a 15 min phagocytosis on ice or at 37°C. Phagocytosis is expressed as % GFP+ neutrophils (A) and GFP relative MFL (4% serum) of the total population (B). Data are the mean of 2 experiments ± SD. [file Image_2.jpeg]

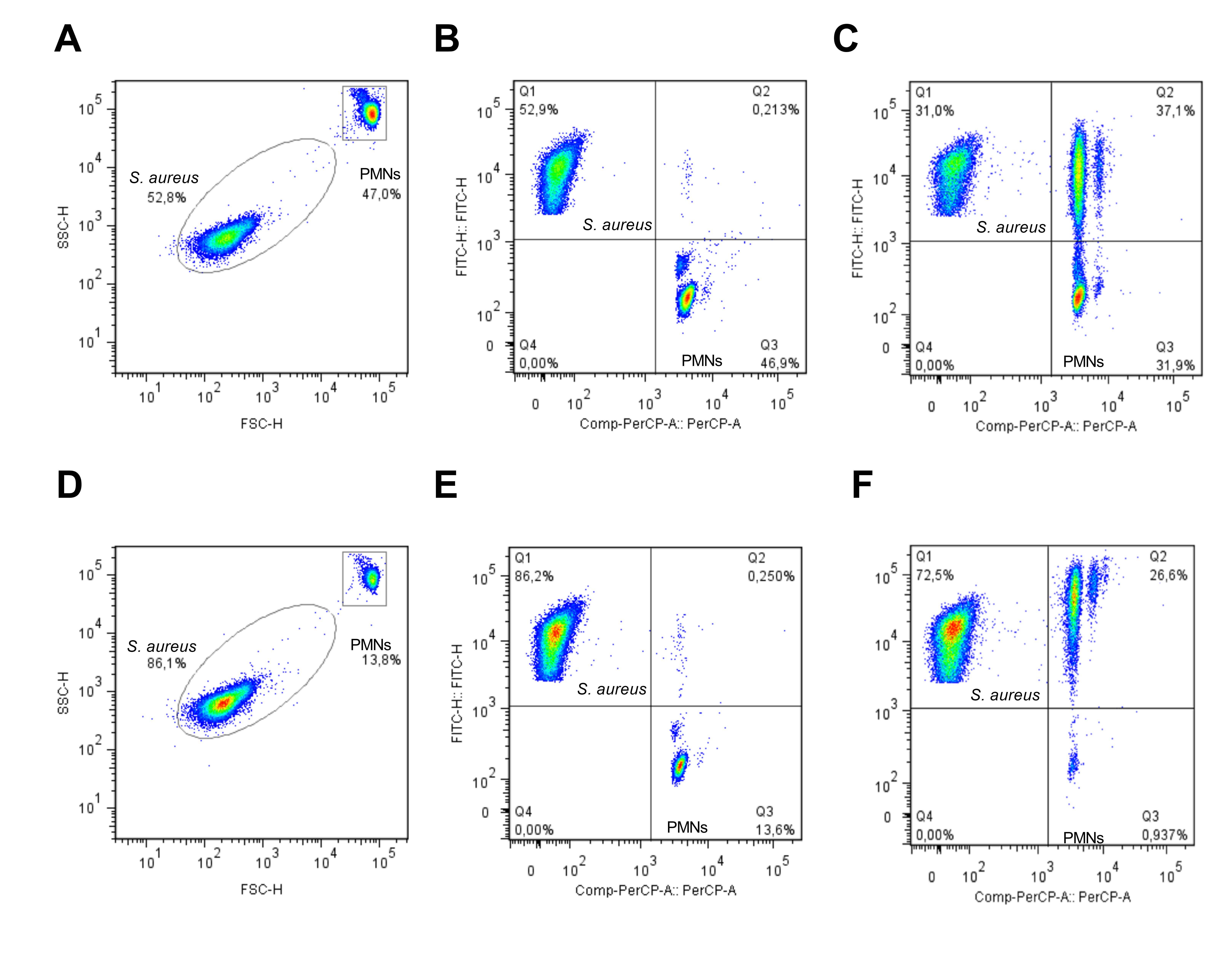

Supplement: Supplementary Figure 3 — Gating strategies for the alternative phagocytosis assay using FITC (or GFP) and LDS-753 (PerCP signal) as thresholod parameters for data aquisition. Shown are representative dot plots for a phagocytosis assays performed with putative bacteria to cell ratio 1:1 (upper panel) and 8:1 (lower panel). During the acquisition, bacteria to cell ratio was verified by gating both bacteria and PMNs by setting logarithmic scatter parameters (panel A for 1:1 and D for 8:1) or fluorescence parameters (panel B for 1:1 and E for 8:1). The actual observed ratio for the 1:1 appeared to be 1.1:1, and for the 8:1 ratio appeared to be 6.3:1, both in agreement for the scatter and the fluorescence based parameters. Dot plots are from the control samples before starting the experiments with 4% NHS, mixed on ice and immediately fixed with paraformaldehyde. Panels C, F are representative fluorescence dotplots for the 15 min phagocytosis timepoint with 4% NHS showing for the 1.1:1 ratio (C) 53.8% GFP+ neutrophils at a 0.45:1 ratio resulting in 59.8% bacterial phagocytosis, and for the 6.3:1 ratio (F) 96.6% GFP+ neutrophils and a 58.2% bacterial phagocytosis. [file Image_3.jpeg]
